# Recurrent pityriasis rosea: A case report

**DOI:** 10.1080/21645515.2017.1409928

**Published:** 2017-12-21

**Authors:** Ang Li, Ping Li, Yanqiong Li, Wenfei Li

**Affiliations:** a Department of Dermatology, Qianfoshan Hospital, Shandong University, Jinan, Shandong, People's Republic of China; b Department of Clinical Medicine, Queen Mary School, Medical School, Nanchang University, Nanchang, Jiangxi, People's Republic of China; c Department of Clinical Medicine, Taishan Medical College, Tai'an, Shandong, People's Republic of China

**Keywords:** hepatitis B vaccine, induce, influenza A (H1N1) vaccine, pityriasis rosea, recurrent

## Abstract

Pityriasis rosea is a papulosquamous skin disorder that occurs most commonly between the ages of 10 and 35 years. Recurrent pityriasis rosea is rare. We report a patient suffering from recurrent pityriasis rosea, whose etiology may be related to either vaccine-induced stimulation of the immune system, or some rare vaccine component(influenza A [H1N1] vaccine, hepatitis B vaccine). We believe that such a case is unique and it has not been reported previously. The patient was successfully treated with a combination of oral cetirizine, a topical steroid cream, and narrowband-ultraviolet B phototherapy. The symptoms of this disorder should be recognized by dermatologists.

## Introduction

Pityriasis rosea is a papulosquamous skin disorder that occurs most commonly between the ages of 10 and 35 years. Classically, it usually begins with a single “herald patch” lesion, followed by the onset of secondary scaly, pinkish skin eruptions within 1–2 weeks. Although its pathogenesis is still not completely understood, a viral infection is likely according to its clinical presentation and immunologic reactions. Pityriasis rosea may inflict substantial discomfort in certain cases. If a women develops pityriasis rosea during pregnancy, it may lead to serious outcomes in newborns, such as low gestational weight, stillbirth, and premature delivery.^1^ Some studies have showed that the approximate incidence of pityriasis rosea is 0.5–2%,^2^ and pityriasis rosea relapses in 3.7% of cases.^3^ Since recurrent pityriasis rosea was first reported in detail by Halkier-Sørensen in 1990, to the best of our knowledge, only a few patients with recurrent pityriasis rosea have been described in English language journals, as determined by a Pubmed search.^4-8^

Here, we report a case of a 32-year-old male who suffered from recurrent pityriasis rosea.

## Patient presentation

**Figure 1.**
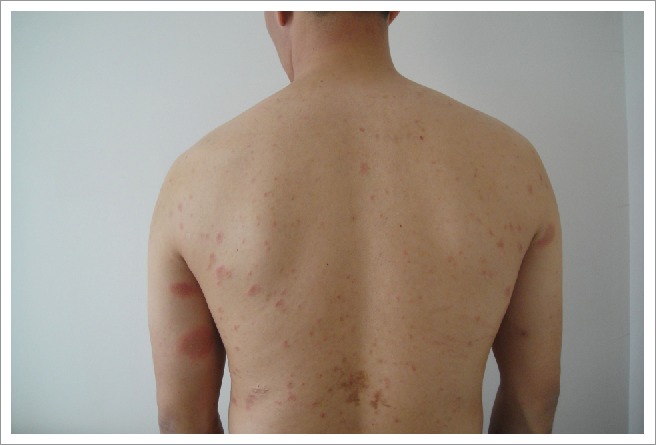
The herald patch on the patient's left upper limb. The herald patch on the inner left upper limb was an approximately 3.5 cm oval, thin plaque with scaly erythema.

**Figure 2.**
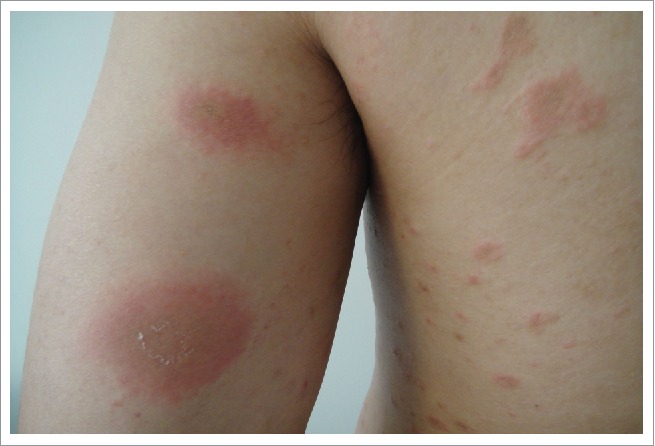
Multiple, erythematosquamous, oval secondary eruptions. Secondary eruptions were multiple, erythematosquamous, oval lesions with a peripheral collarette scale, and they were distributed along the lines of cleavage and gave a “Christmas tree” pattern on the patient's trunk.

A 32-year-old Chinese male complained of mildly pruritic rashes for 20 days. The patient had an influenza A (H1N1) vaccine (Split Virion) (VAXIGRIP, Shenzhen Sanofi Pasteur Biological Products Company Limited, Shenzhen, People's Republic of China) injection 11 days prior to the onset of the initial rash. He casually found a herald skin lesion on his left upper limb 3 days after he received an inactivated influenza A (H1N1) vaccine in May 2016. With his skin disorder progressing, he felt a mild pruritus. Secondary eruptions appeared on the neck, trunk, and extremities, and they increased in number. However, there were no eruptions on the abdomen or buttocks. Compared with other patients suffering from pityriasis rosea, his rashes were larger. Most of the lesions were distributed on the neck, chest, back, and proximal upper limbs. Physical examination showed a herald patch on his inner left upper limb, and the lesion was an approximately 3.5-cm oval, thin plaque with scaly erythema (Fig. 1). The secondary eruptions were multiple, erythematosquamous, oval lesions with a peripheral collarette scale; they were distributed along the lines of cleavage, and gave a “Christmas tree” pattern on the patient's trunk (Fig. 2).

Laboratory analysis revealed the patient's full blood count was normal, and a normal erythrocyte sedimentation rate of 12 mm/h (normal: 0–15 mm/h) was observed; serological examination revealed that he was negative for syphilis and antibodies to human immunodeficiency virus, and microscopic examination showed that the patient did not exhibit any signs of fungal infection. The patient's C-reactive protein (CRP) level was 16.2 mg/L (normal 0–3 mg/L), and he was negative for anti-nuclear antibody and anti-neutrophil cytoplasmic antibodies. He had no history of food allergies, and he denied smoking or suffering from alcohol abuse. He did not receive any medication before the influenza A (H1N1) vaccine injection.

**Figure 3.**
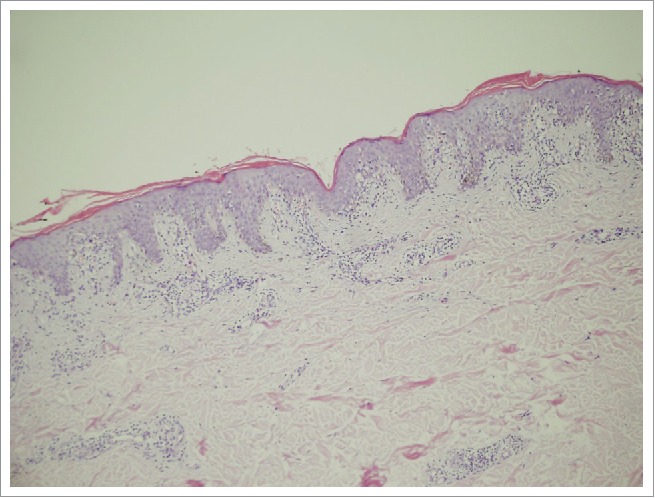
Slight hyperkeratosis and mild intercellular edema. Slight hyperkeratosis and a reduced granular cell layer. A mild intercellular edema in the superficial dermis (hematoxylin and eosin (H&E) stain, × 100).

**Figure 4.**
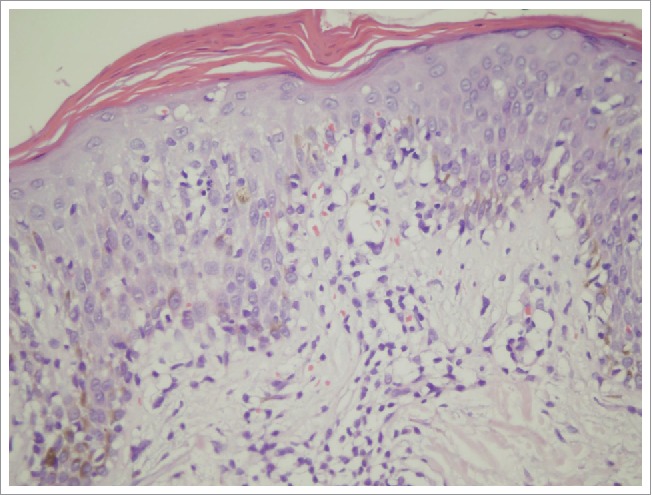
The infiltration of lymphocytes into the epidermis and extravascular erythrocytes into the superficial dermis. The epidermis showed slight hyperkeratosis, parakeratosis, a mild intercellular edema, and the infiltration of a few lymphocytes. There was a mild infiltration of perivascular lymphocytes and extravascular erythrocytes into the superficial dermis (hematoxylin and eosin (H&E), × 400).

A histopathological biopsy taken from the border of the larger patch showed slight hyperkeratosis and a reduced granular cell layer. There was a mild intercellular edema in the superficial dermis (Fig. 3). The epidermis showed slight hyperkeratosis, parakeratosis, and a mild intercellular edema, as well as the infiltration of a few lymphocytes. There was a mild infiltration of perivascular lymphocytes and extravascular erythrocytes in the superficial dermis (Fig. 4). Based on the clinical and histopathological features, a final diagnosis of pityriasis rosea was put forth.

The patient was treated with oral cetirizine (Shidi, Qilu Pharmaceutical Company Limited, Jinan, People's Republic of China) to ease allergic symptoms, such as pruritus, a topical steroid cream(Ailuosong, Shanghai Schering-plough Pharmaceutical Company Limited, Shanghai, People's Republic of China), and narrowband-ultraviolet B phototherapy. His symptomatic itching gradually disappeared, and the lesions resolved within 12 days.

14 months later, the patient came to our clinic again presenting multiple, erythematosquamous, oval lesions. His rashes occurred 5 days after the patient received a hepatitis B vaccine (Engerix B, GlaxoSmithKline, Brentford, England) injection in July 2017. The eruptions had similar characteristics, such as a peripheral, collarette scale along the lines of cleavage, and they were distributed on the neck, chest, back, and proximal upper limbs. Physical examination revealed a herald patch on his left back, and it was 4.1-cm oval, thin plaques. The patient received the same treatment, and his rashes gradually vanished within 2 weeks. We made the diagnosis of recurrent pityriasis rosea.

## Discussion

Pityriasis rosea is a clinical entity that was named by Gibert in 1860. It is a sudden-onset, self-limiting, and papulosquamous disease. The exact etiology of pityriasis rosea is still not clear, but many factors, such as viruses, bacteria, fungi, and non-infective etiologies, may induce pityriasis rosea. Recent evidence suggests that reactivation of HHV-6 and HHV-7 may contribute to the development of the lesions,^9^ and some drugs, for example acyclovir, as well as biological agents,^10^ have been reported to be associated with pityriasis rosea. Some cases also developed pityriasis rosea following human papillomavirus vaccination and pneumococcal vaccine.^11^^,^^12^

Only a few reports about recurrent pityriasis rosea are available. One report showed that recurrent pityriasis rosea is associated with coryzal illness and fever,^6^ and another showed recurrent pityriasis rosea associated with oral ulcers,^7^ while others found no explanation for pityriasis rosea recurrences.^4^^,^^5^^,^^8^

A vaccine can provide active acquired immunity to a particular disease, and the WHO advises vaccination for all individuals ≥ 9 months living in countries or areas at risk.^13^ The influenza and hepatitis B vaccines are very well tolerated and associated with only very low rates of adverse reactions.^14^ Lichenoid drug reactions to vaccinations are very rare, yet one case developed a lichenoid drug eruption following vaccination against the influenza virus.^15^ With respect to the recurrence pityriasis rosea in our patient, one recurrence followed influenza A (H1N1) vaccine and another was associated with the hepatitis B vaccine. The patient had no history of food allergies and denied taking medications, and he was negative for syphilis and antibodies to human immunodeficiency virus, and a microscopic examination revealed that he was negative for a fungal infection. Thus, we speculate that either vaccine-induced stimulation of the immune system or some rare vaccine component might have contributed to the etiology of pityriasis.

Our patient differs from a patient with recurrent pityriasis rosea who was described previously.^4-8^ First, his rashes were associated with vaccine injection. We think that either vaccine-induced stimulation of the immune system or some rare vaccine component might have led to the skin disorder. Second, his rashes were larger than those commonly found on patients with pityriasis rosea. This may be associated with stronger immune reactions, compared with some patients; Third, his eruptions were distributed on the neck, chest, back, and extremities, but no eruptions were present on the abdomen and buttocks. To the best of our knowledge, such a case has not been described in the English language literature.

In summary, we have described a case of recurrent pityriasis rosea. This case represents a rare report of recurrent pityriasis rosea, the etiology may be related to either vaccine-induced stimulation of the immune system, or some rare vaccine component. We believe such a case is unique and it has not been reported previously. The patient was successfully treated with a combination of oral cetirizine, a topical steroid cream, and narrowband-ultraviolet B phototherapy. The symptoms of this disorder should be recognized by dermatologists.
